# Value of perfusion parameters histogram analysis of triphasic CT in differentiating intrahepatic mass forming cholangiocarcinoma from hepatocellular carcinoma

**DOI:** 10.1038/s41598-021-02667-4

**Published:** 2021-11-30

**Authors:** Fang Zhao, Guodong Pang, Xuejing Li, Shuo Yang, Hai Zhong

**Affiliations:** 1 Department of Radiology, The Second Hospital, Cheeloo College of Medicine, Shandong University, No.247, Beiyuan Road, Tianqiao District, Jinan, 250033 Shandong China; 2 Department of Radiology, Qilu Hospital, Cheeloo College of Medicine, Shandong University, Jinan, 250012 China; 3 Jinan Blood Center, Jinan, 250001 Shandong China

**Keywords:** Cancer, Diseases, Gastroenterology, Oncology, Mathematics and computing

## Abstract

We aim to gain further insight into identifying differential perfusion parameters and corresponding histogram parameters of intrahepatic mass-forming cholangiocarcinoma (IMCC) from hepatocellular carcinomas (HCCs) on triphasic computed tomography (CT) scans. 90 patients with pathologically confirmed HCCs (n = 54) and IMCCs (n = 36) who underwent triple-phase enhanced CT imaging were included. Quantitative analysis of CT images derived from triphasic CT scans were evaluated to generate liver perfusion and histogram parameters. The differential performances, including the area under the receiver operating characteristic curve (AUC), specificity, and sensitivity were assessed. The mean value, and all thepercentiles of the arterial enhancement fraction (AEF) were significantly higher in HCCs than in IMCCs. The difference in hepatic arterial blood supply perfusion (HAP) and AEF (ΔHAP = HAP_tumor _− HAP_liver_, ΔAEF = AEF_tumor _− AEF_liver_) for the mean perfusion parameters and all percentile parameters between tumor and peripheral normal liver were significantly higher in HCCs than in IMCCs. The relative AEF (rAEF = ΔAEF/AEF_liver_), including the mean value and all corresponding percentile parameters were statistically significant between HCCs and IMCCs. The 10th percentiles of the ΔAEF and rAEF had the highest AUC of 0.788 for differentiating IMCC from HCC, with sensitivities and specificities of 87.0%, 83.3%, and 61.8%, 64.7%, respectively. Among all parameters, the mean value of ∆AEF, the 75th percentiles of ∆AEF and rAEF, and the 25th percentile of HF_tumor_ exhibited the highest sensitivities of 94.4%, while the 50th percentile of rAEF had the highest specificity of 82.4%. AEF (including ΔAEF and rAEF) and the corresponding histogram parameters derived from triphasic CT scans provided useful value and facilitated the accurate discrimination between IMCCs and HCCs.

## Introduction

Intrahepatic cholangiocarcinoma (ICC), originating from the epithelial cells of the bile duct, is the second most common primary cancer of the liver after hepatocellular carcinoma (HCC), and its incidence and mortality have been increasing in recent decades^1,2^. ICC can be divided into three types according to morphology, including intraductal growing, periductal infiltrating, and mass forming. Among the three types, intrahepatic mass forming cholangiocarcinoma (IMCC) is the most common form^3,4^. IMCC has similar risk factors with HCC, including chronic viral hepatitis, cirrhosis, and so on^5,6^, but has distinctly different prognosis and different treatment methods^7^. For HCC, percutaneous ablation, radiofrequency ablation, surgical resection and liver transplantation are all available treatment options, while thorough surgical resection with negative margins is the only way to cure IMCC^8,9^. Therefore, accurate preoperative differentiation of IMCC from HCC is avital clinical issue for overcoming such cancers.

Previous studies had shown that the differentiation between IMCCs and HCCs can be made based on MR or CT imaging feature analysis and clinical findings^10–12^. In clinical practice, HCC typically shows intense hyper enhancement on the arterial phase, followed by washout during dynamic imaging. Conversely, IMCC typically shows peripheral enhancement in the arterial phase, with centripetal progressive reinforcement on delayed phase^12^. The accuracy of these techniques depends on the size of the tumors, complications of cirrhosis, and the experience of the radiologist^13,14^. IMCC in cirrhotic patients may be hypervascular on the arterial phase images due to the increasing density of the arteries and microvessels in cirrhosis and precirrhotic liver, thus, exhibiting overlapping phenotypes with the appearance of typical HCC^15,16^. Approximately 10–20% of HCCs may exhibit hypoenhancement in the arterial phase owing to the insufficient development of tumor neovascularity and the retention of dual blood supply^17^; thus, mimicking IMCC^10^.

For patients with hepatic tumors, accurate evaluation of the hemodynamic blood status, especially in the area of hepatic perfusion, could provide vital information for prognosis assessments and appropriate clinical treatment options. The proportion of hepatic artery and portal vein blood supply varies according to the pathological changes of the liver. Liver cancers differ in their type of vascularization^18^ and in principle, HCC is hyper-vascular and initially vascularized by the hepatic artery. In contrast, IMCC contains a large amount of fibrous stroma, less blood supply, and a slower clearance rate of contrast agents than HCC.

As a functional vascular imaging technique, CT perfusion imaging could be used to monitor the hemodynamic status of tumors. Liver perfusion computed tomography (PCT) could be used to acquire precise blood flow values of liver diseases and can quantitatively measure perfusion parameters. Traditional PCT is largely unused clinically attributed to the high radiation dose and poor image quality. However, standard triphasic CT using the dual maximum slope model, which was first proposed by Blomley et al.^19^, can quantitatively obtain a series of perfusion parameters which could be used to assess the tumor blood supply status. To our knowledge, no previous study has illustrated the perfusion parameters or histogram parameters originated from triphasic CT enhancement scans to distinguish HCC from IMCC.

Thus, the purpose of this study was to explore whether different perfusion parameters and corresponding histogram parameters could provide additional value to triphasic CT scans in differentiating IMCCs from HCCs. The optimal parameters for differentiation were also determined.

## Materials and methods

This retrospective study was approved by the ethics committee of the Second Hospital of Shandong University. All of the methods were performed in accordance with the 1975 declaration of Helsinki and corresponding guidelines. Patient informed consent was waived because this study was a retrospective study. Waiver for informed consent was approved by the Ethics Committee of The Second Hospital of Shandong University.

### Patient selection

**Figure 1:**
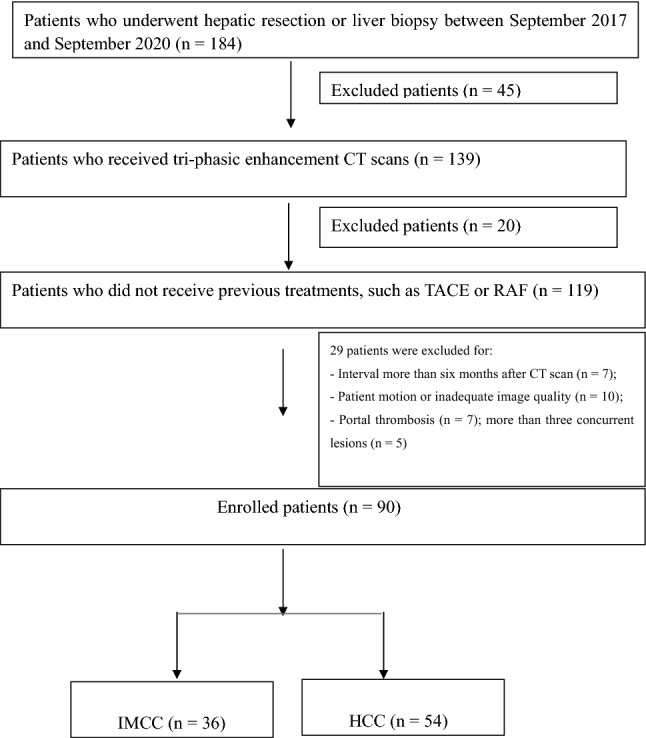
Flow diagram detailing the inclusion and exclusion criteria for the study.

We consulted the electronic medical records of our hospital from September 2017 to September 2020. The inclusion criteria were as follows: patients that (a) underwent traditional triphasic CT scans with adequate image quality and without artefacts; (b) had no history of prior treatment of hepatic tumor; and (c) had histologically confirmed IMCCs or HCCs according to the 2010 World Health Organization classification^20^ within 6 months of the CT scans. A flow diagram for the study population is presented in Fig. 1.

### Imaging techniques

In all patients, scanning was performed using a Discovery 750HD CT scanner (GE Healthcare, Waukesha, WI, USA) with the following parameters: tube voltage: 120 kV, tube current 250 mA, collimation: 0.625 mm, a rotation time of 0.5 s, with the slice thickness: 5 mm, and a gap of 2 mm. Iodinated contrast agent (Omnipaque 370 mg iodine/mL, GE) followed by a 30 mL saline chaser was injected into an antecubital vein at a rate of 3.5–4.0 mL/s with power injector 1.5 mL/kg. Scan delay for the arterial phase, portal venous phase and delayed phase was 30–35 s, 60–70 s, 180 s.

### Imaging analysis and perfusion parameter measurements

The Digital Imaging and Communications in Medicine (DICOM) files of dynamic enhanced CT data were processed with CT hemodynamic kinetics software (CT Kinetics, GE Healthcare). Tumor regions of interest (ROIs) were delineated along the margins of tumor lesion on all continuous sections, including any cystic, necrotic, and hemorrhagic portions. If multiple lesions were present in the liver, the largest lesion confirmed by pathology was selected to delineate the regions of interest. Tumor-free ROIs with the same size as the tumor were drawn in the same lobe of normal liver avoiding large blood vessels. The perfusion parameters of hepatic arterial supply perfusion (HAP), portal vein blood supply perfusion (PVP), and arterial enhancement fraction (AEF) were measured using CT hemodynamic kinetics software on the basis of the model-free maximum method. Measurements were performed by two independent radiologists (F.Z and H.Z with 6 and 15 years of experience in abdominal imaging, respectively). AEF was defined as the ratio of the absolute increment of attenuation during the arterial phase to the absolute increment of attenuation during the portal venous phase^21^. The other perfusion parameters were also calculated, including total HF (HF_tumor_ = HAP_tumor_ + PVP_tumor)_, total HF (HF_liver_ = HAP_liver_ + PVP_liver_), the differences in flow between tumor and liver (ΔHF = HF_tumor_ − HF_liver_), relative flow (rHF = ΔHF/HF_liver_), the difference in HAP (ΔHAP = HAP_tumor_ − HAP_liver_), relative HAP (rHAP = ΔHAP/HAP_liver_), the difference in PVP (ΔPVP = PVP_tumor_ − PVP_liver_), relative PVP (rPVP = ΔPVP/PVP_liver_), the difference in AEF (ΔAEF = AEF_tumor_ − AEF_liver_), and the relative AEF (rAEF = ΔAEF/AEF_liver_). From these voxel-by-voxel HAP, PVP, and AEF values, a histogram analysis for each lesion was processed, including the median value, mean value, standard deviation (SD), 10th to 90th percentiles, variance, skewness, and kurtosis^21–23^.

### Statistical analysis

All Statistical analyses were performed using SPSS version 19.0 (SPSS statistics; IBM). Intraclass correlation analysis was applied to assess interobserver agreement between the two radiologists using an intraclass correlation coefficient (ICC). ICC values under 0.4, between 0.4 to 0.8, and above 0.8 indicated weak, moderate, and strong agreement respectively. If there was a disagreement between observers, an agreement was achieved through discussion. The Kolmogorov–Smirnov test was first used for normally-distributed perfusion parameters and histogram parameters. Then, quantitative data were compared using an independent Student’s *t-*test or Mann–Whitney U-test between IMCC and HCC. The potential diagnostic performance of each parameter in differentiating IMCC from HCC was determined by means of receiver operating characteristic (ROC) curve analyses. Next, the cutoff values, the sensitivity and specificity at the threshold values for each parameter were determined. Diagnostic differences in the sensitivity and specificity between single parameters or the combination of two parameters were compared using the McNemar test. A two-tailed *P* < 0.05 indicated statistical significance.

### Ethical approval

This retrospective study was approved by the ethics committee of the Second Hospital of Shandong University. All of the methods were performed in accordance with the 1975 declaration of Helsinki and corresponding guidelines. Patient informed consent was waived because this study was a retrospective study.

## Results

### Patient characteristics

Finally, 36 patients diagnosed with IMCCs (mean age, 58.9 years; male:female = 20:16) and 54 patients diagnosed with HCCs (mean age, 57.3 years; male:female = 36:18) were included. Pathological diagnoses were derived from all patients via surgical specimens (n = 68; HCCs [n = 44], IMCCs [n = 24]) or percutaneous liver biopsy (n = 22; HCCS [n = 10], IMCCs [n = 12]). The mean interval between the CT imaging and surgery or biopsy was 5.5 days (range 1–19 days) for patients with IMCCs, and 7 days (range 1–43 days) for patients with HCCs. In the HCC group, 44 patients (44/54; 81.5%) had HBV or HCV infection and 10 patients (10/54; 18.5%) had alcoholic liver cirrhosis. In the IMCC group, five patients (5/36; 14%) had HBV or HCV infection and four patients (4/36; 11%) had alcoholic liver cirrhosis. There was no significant difference in the age, sex, or tumor size between HCC and IMCC patients. According to the liver function based on the Child–Pugh classification, there were 38 patients in the Child–Pugh class A group and 25 patients in the Child–Pugh class B/C group. Patients with IMCC had lower a-fetoprotein (AFP) levels than those with HCC(*P* < 0.001), and the IMCC group had fewer cases of liver cirrhosis than the HCC group (*P* < 0.001). Patient characteristics are summarized in Table 1.

**Table 1:** Clinical characteristics of patients with IMCC and HCC.

	IMCC(n = 36)	HCC(n = 54)	*P* Value
Age (years), mean ± SD	58.9 ± 10.0	57.3 ± 11.3	0.169
Male:Female	20:16	36:18	0.242
Size (mm), mean ± SD	52.1 mm ± 23.8	54.5 mm ± 27.2	0.876
**Background liver**			< 0.001
Fibrosis	2	18	
Cirrhosis	7	36	
**Etiology**			< 0.001
Hepatitis B	3	30	
Hepatitis C	2	14	
Alcoholism	4	10	
**Child–Pugh classification**			< 0.001
A	7	39	
B/C	2	15	
AFP Level(ng/mL)	7.3(2.7–1031.0)	72.5(10.1–5050.0)	< 0.001

*IMCC* intrahepatic mass-forming cholangiocarcinoma, *HCC* hepatocellular carcinoma, *SD* standard deviation, *AFP* a-fetoprotein.

### Inter-observer agreement

To evaluate interobserver agreement for perfusion parameters and corresponding histogram parameters analyses, the quadratic weighted *k* statistics were calculated and exhibited excellent interobserver agreement (*k* = 0.87). Hence, the quantitative perfusion and corresponding histogram analysis were used for the subsequent analyses.

### Tumor perfusion parameters and corresponding histogram parameters between IMCCs and HCCs

**Figure 2:**
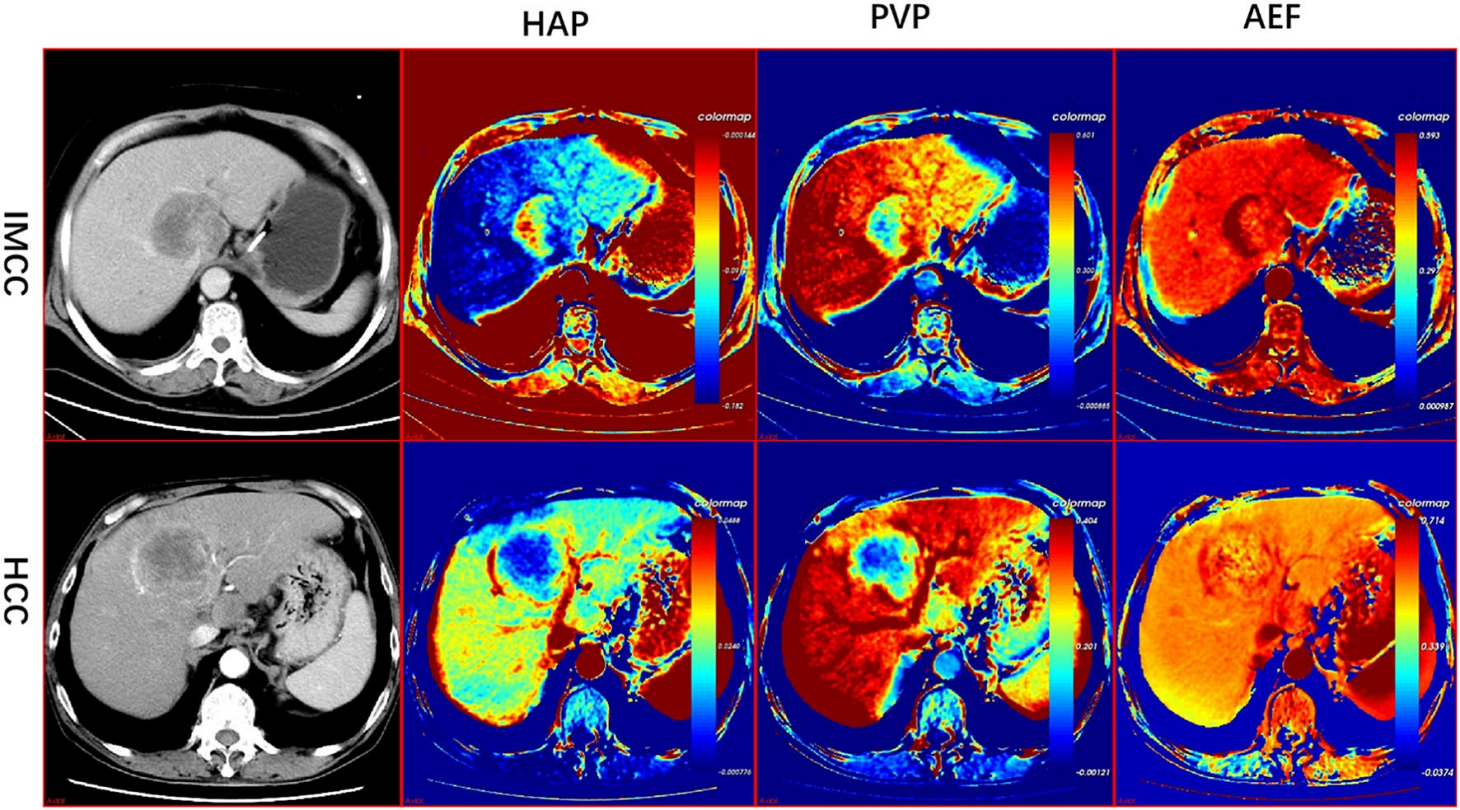
Traditional enhancement image and pharmacokinetic images of IMCC and HCC. For the patients with IMCC, the HAP image showed high perfusion in the margin and relatively low perfusion in the center. PVP images showed hyperperfusion from the peripheral to the central part of the tumor. For patients with HCC, the HAP image showed high perfusion in the rim, while the PVP image showed homogeneous low perfusion in the complete lesion. The AEF images both showed heterogeneous high perfusion for two tumor lesions. *HAP* Hepatic arterial supply perfusion, *PVP* Portal venous supply perfusion, *AEF* Arterial enhancement fraction, *IMCC* Intrahepatic mass-forming cholangiocarcinoma, *HCC* Hepatocellular Carcinoma.

The data of the perfusion parameters and histogram parameters were non-normally distributed. Therefore, the Mann–Whitney *U* test was performed for data analysis. The perfusion parameters for IMCCs and HCCs are shown in Table 2. Representative CT perfusion images derived from the triphasic CT scans of IMCCs and HCCs are shown in Fig. 2. The AEF mean value, the difference in AEF between tumor and normal liver (ΔAEF = AEF_tumor_ − AEF_liver_), and the relative AEF (rAEF = ΔAEF/AEF_liver_) were significantly higher in HCCs than in IMCCs (*P* ≤ 0.001). The mean value of HAP in patients with HCC was significantly higher than in patients with IMCCs (*P* = 0.024). There were no statistical differences in the other perfusion parameters between IMCCs and HCCs (*P* > 0.05).

**Table 2:** Liver perfusion parameters and histogram parameters for patients with IMCCs and HCCs.

Group	Mean Value		± SD		*P Value*
	HCCs (n = 54)	IMCCs(n = 36)	HCCs (n = 54)	IMCCs (n = 36)	
HAP (mean)	− 0.001	− 0.014	0.049	0.042	0.059
HAP (0.1)	− 0.018	− 0.034	0.057	0.059	0.062
HAP (0.25)	− 0.010	− 0.024	0.0532	0.051	0.072
HAP (0.5)	− 0.001	− 0.013	0.049	0.041	0.065
HAP (0.75)	0.007	− 0.002	0.044	0.035	0.064
HAP (0.9)	0.015	0.006	0.041	0.033	0.119
PVP (mean)	0.261	0.254	0.076	0.096	0.733
PVP (0.1)	0.188	0.155	0.077	0.094	0.079
PVP (0.25)	0.221	0.197	0.076	0.096	0.182
PVP (0.5)	0.260	0.250	0.077	0.099	0.609
PVP (0.75)	0.299	0.306	0.080	0.109	0.759
PVP (0.9)	0.335	0.356	0.084	0.122	0.389
AEF (mean)	0.594	0.536	0.124	0.120	0.001*
AEF (0.1)	0.511	0.420	0.089	0.116	< 0.001*
AEF (0.25)	0.548	0.475	0.091	0.090	< 0.001*
AEF (0.5)	0.587	0.518	0.107	0.083	< 0.001*
AEF (0.75)	0.630	0.567	0.140	0.080	0.005*
AEF (0.9)	0.678	0.616	0.198	0.102	0.040*
HAP(variance)	0.0002	0.0005	0.0003	0.002	0.607
HAP(skewness)	11.272	− 0.028	82.728	0.661	0.939
HAP(kurtosis)	130.846	3.674	936.972	1.360	0.329
PVP(variance)	0.004	0.008	0.003	0.010	0.009*
PVP(skewness)	6.122	0.257	44.687	0.645	0.121
PVP(kurtosis)	62.578	3.794	433.540	1.334	0.355
AEF(variance)	0.018	0.061	0.044	0.207	0.113
AEF(skewness)	1.597	3.295	4.107	7.032	0.435
AEF(kurtosis)	35.482	105.111	96.997	271.455	0.033*

*Statistically significant difference between the two groups (P < 0.05).

*HAP* hepatic artery perfusion (mL/100 mL/min), *PVP* portal vein perfusion (mL/100 mL/min), *AEF* arterial enhancement fraction (%), *IMCC* intrahepatic mass-forming cholangiocarcinoma, *HCC* hepatocellular carcinoma.

The histogram parameters for IMCCs and HCCs are shown in Tables 2 and 3. All of the percentiles of the AEF mean value, ΔAEF, and rAEF were significantly higher in patients with HCCs than with IMCCs (*P* < 0.05). The 10th, 25th, 50th, and 75th percentiles of HAP were significantly higher in HCCs than in IMCCs (*P* < 0.05). The kurtosis of AEF was higher in patients with HCCs than with IMCCs (*P* = 0.033), and the variance of PVP was lower in HCCs than in IMCCs (*P* = 0.009). The 25th percentile of HF_tumor_ was significantly higher in patients with HCCs than IMCCs (*P* = 0.036). The 10th and 25th percentiles of ∆HF were also higher in HCCs than in IMCCs (*P* = 0.011 and 0.030, respectively). For the other histogram parameters, there were no statistically significant differences between the two groups.

**Table 3:** The parameters of ∆HAP, rHAP, ∆PVP, rPVP, ∆AEF, rAEF, HF, ∆HF, rHF, and their corresponding percentiles in patients with IMCCs and HCCs.

Group	Mean value		± SD		*P* Value
	HCCs (n = 54)	IMCCs (n = 36)	HCCs (n = 54)	IMCCs (n36)	
∆HAP (mean)	0.016	0.008	0.029	0.035	0.024*
∆HAP (0.1)	0.007	− 0.006	0.027	0.030	0.009*
∆HAP (0.25)	0.011	0.002	0.027	0.029	0.020*
∆HAP (0.5)	0.016	0.009	0.029	0.034	0.028*
∆HAP (0.75)	0.020	0.016	0.031	0.044	0.046*
∆HAP (0.9)	0.024	0.021	0.033	0.051	0.076
∆PVP (mean)	− 0.083	− 0.104	0.097	0.130	0.391
∆PVP (0.1)	− 0.122	− 0.173	0.112	0.136	0.058
∆PVP (0.25)	− 0.103	− 0.145	0.104	0.135	0.109
∆PVP (0.5)	− 0.083	− 0.107	0.099	0.131	0.325
∆PVP (0.75)	− 0.062	− 0.068	0.093	0.138	0.807
∆PVP (0.9)	− 0.045	− 0.034	0.091	0.146	0.674
∆AEF (mean)	0.093	0.028	0.121	0.125	< 0.001*
∆AEF (0.1)	0.038	− 0.068	0.084	0.124	< 0.001*
∆AEF (0.25)	0.061	− 0.022	0.087	0.101	< 0.001*
∆AEF (0.5)	0.087	0.011	0.102	0.093	< 0.001*
∆AEF (0.75)	0.116	0.048	0.135	0.090	0.001*
∆AEF (0.9)	0.152	0.087	0.194	0.110	0.012*
rHAP (mean)	− 3.493	0.779	30.734	0.912	0.986
rHAP (0.1)	0.721	1.199	0.935	1.608	0.377
rHAP (0.25)	0.677	1.659	1.369	4.352	0.482
rHAP (0.5)	2.350	0.772	12.070	0.774	0.706
rHAP (0.75)	− 4.061	0.748	38.382	0.768	0.725
rHAP (0.9)	1.168	0.700	5.107	0.956	0.662
rPVP (mean)	0.805	0.749	0.374	0.308	0.837
rPVP (0.1)	0.747	0.514	1.017	0.327	0.143
rPVP (0.25)	0.749	0.616	0.510	0.320	0.258
rPVP (0.5)	0.806	0.737	0.386	0.313	0.675
rPVP (0.75)	0.863	0.857	0.309	0.323	0.532
rPVP (0.9)	0.910	0.953	0.269	0.344	0.258
rAEF (mean)	1.189	1.056	0.219	0.238	< 0.001*
rAEF (0.1)	1.082	0.867	0.176	0.248	< 0.001*
rAEF (0.25)	1.128	0.963	0.170	0.198	< 0.001*
rAEF (0.5)	1.177	1.028	0.186	0.181	< 0.001*
rAEF (0.75)	1.230	1.099	0.232	0.174	0.001*
rAEF (0.9)	1.293	1.171	0.321	0.209	0.011*
HF (0.25)	0.211	0.173	0.073	0.098	0.036*
∆HF (0.1)	− 0.115	− 0.179	0.107	0.120	0.011*
∆HF (0.25)	− 0.092	− 0.143	0.098	0.116	0.030*

*Statistically significant difference between the two groups (P < 0.05).

*∆HAP* Difference in hepatic arterial perfusion (HAP_tumor_−HAP_liver_), *∆HF* Difference in blow between tumor and liver (HF_tumor_−HF_liver_), *∆PVP* Difference in portal vein perfusion (PVP_tumor_−PVP_liver_), *∆AEF* Difference in the arterial enhancement fraction (AEF_tumor_−AEF_liver_), *rHAP* Relative hepatic arterial perfusion (∆HAP/HAP_liver_), *rHF* Relative total tumor flow (∆HF/HF_liver_), *rPVP* Relative portal vein perfusion (∆PVP/PVP_liver_), *rAEF* Relative arterial enhancement fraction (∆AEF/AEF_liver_), *IMCC* Intrahepatic mass-forming cholangiocarcinoma, *HCC* Hepatocellular carcinoma.

### Differential diagnostic ability of the perfusion parameters and the corresponding histogram parameters for IMCCs and HCCs

**Figure 3:**
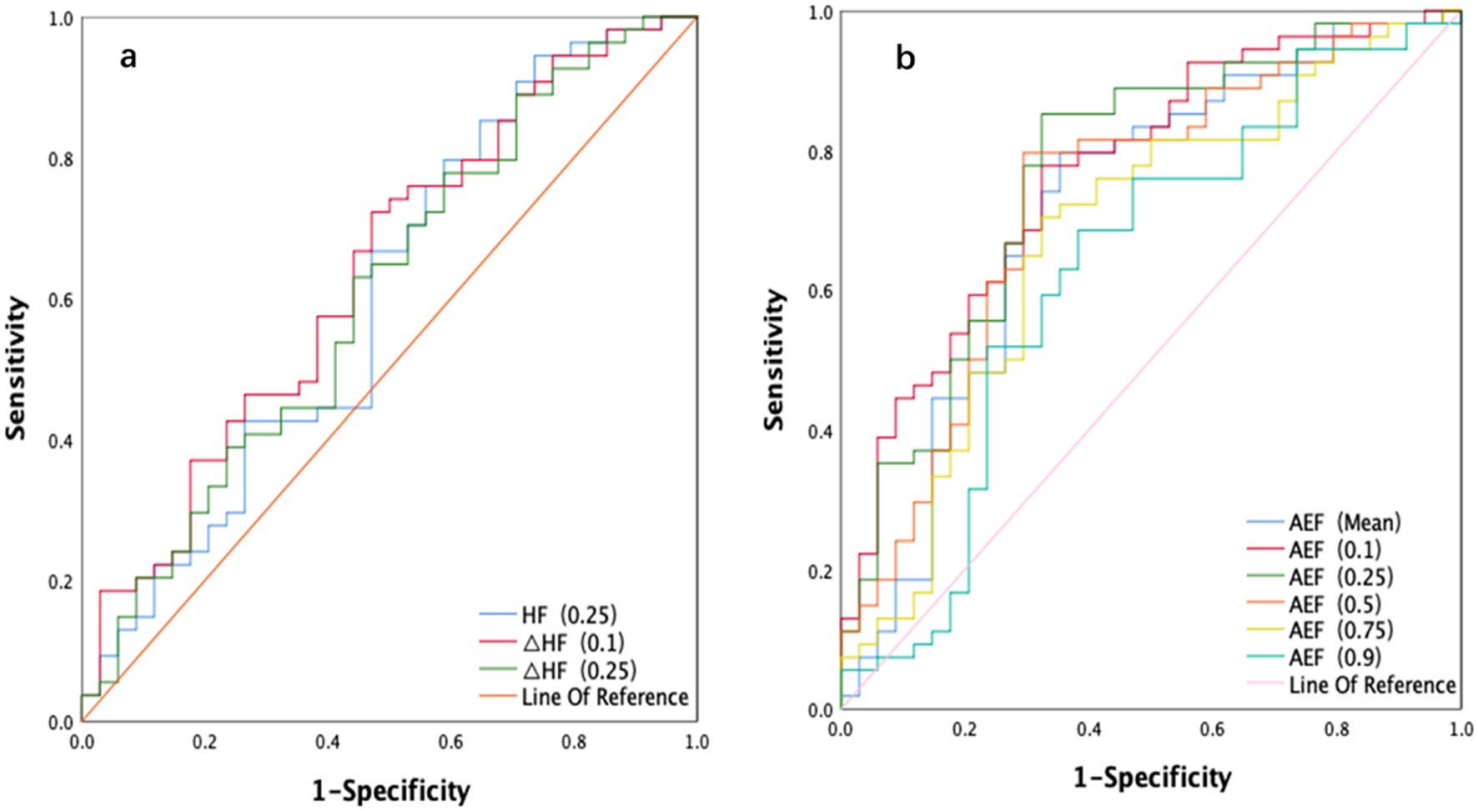
The ROC analysis of HF, AEF, and corresponding percentiles of the parameters for IMCC and HCC (3a and 3b). *HF* Hepatic blood flow, *AEF* Arterial enhancement fraction, *ROC* Receiver operating characteristic, *IMCC* Intrahepatic mass-forming cholangiocarcinoma, *HCC* Hepatocellular Carcinoma.

**Figure 4:**
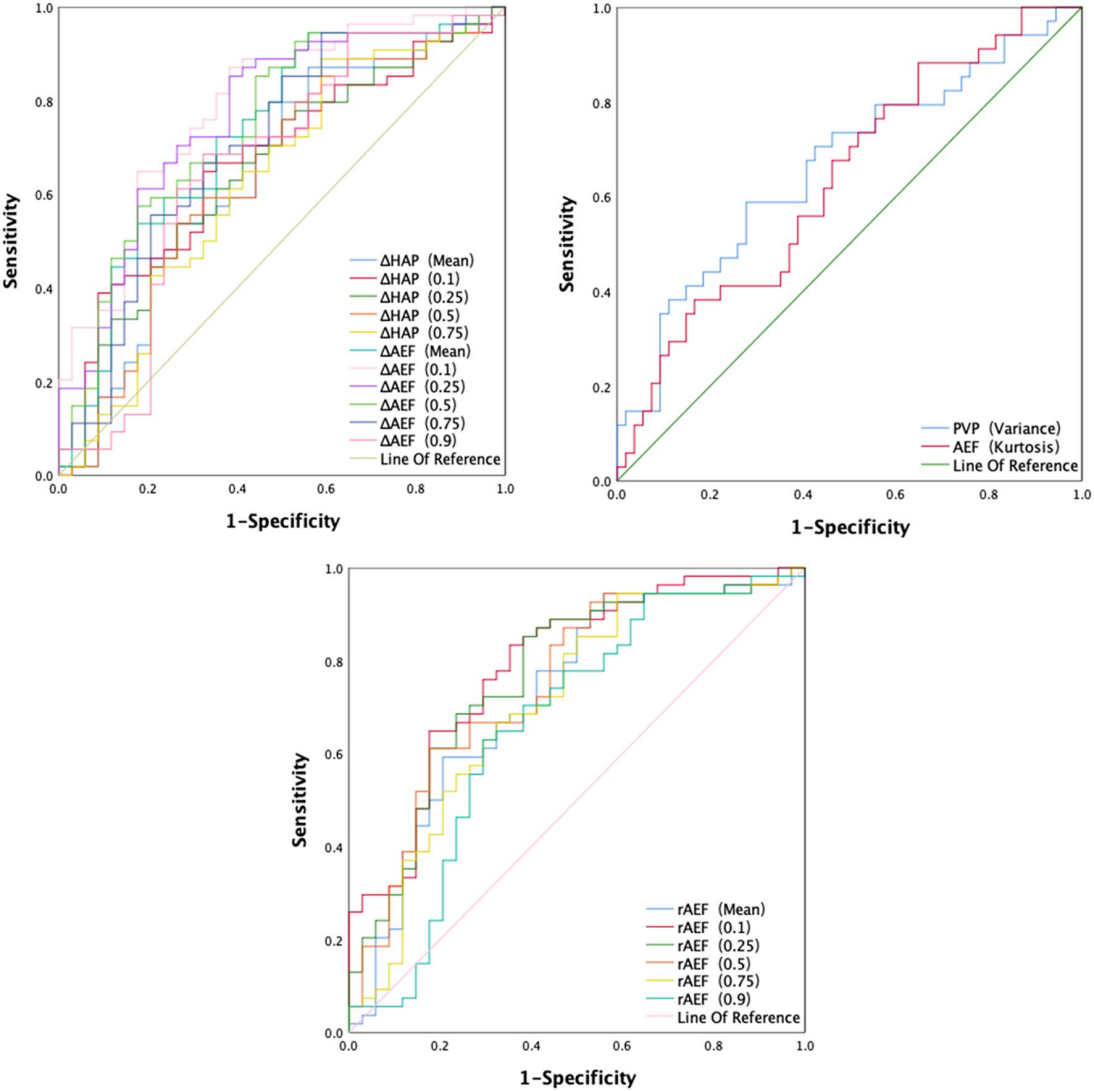
The ROC analysis of the ΔAEF, rAEF, ΔHAP, and the corresponding percentiles, PVP_variance_, AEF_kurtosis_for IMCC and HCC. *ΔAEF* Difference in arterial enhancement fraction (AEF_tumor_−AEF_liver_), *rAEF* Relative arterial enhancement fraction (ΔAEF/AEF_liver_), *ΔHAP* Difference in hepatic arterial perfusion (HAP_tumor_−HAP_liver_), *PVP*_*variance*_ The variance of the portal vein perfusion, *AEF*_*kurtosis*_ The kurtosis value of the arterial enhancement fraction, *ROC* Receiver operating characteristic, *IMCC* Intrahepatic mass-forming cholangiocarcinoma, *HCC* Hepatocellular carcinoma.

ROC curves were used to evaluate the ability of the statistically significant tumor perfusion parameters and corresponding histogram parameters to discriminate between IMCCs and HCCs. As shown in Figs. 3 and 4, and Table 4, of the mean value of AEF and all corresponding percentiles of the histogram analysis, the 10th percentile of the AEF had the highest value of 0.769. The sensitivity and specificity were also 77.8% and 67.6%, respectively, with a cutoff value of 0.466. The positive predictive value (PPV) and negative predictive value (NPV) were 0.792 and 0.657, respectively. The 25th percentile of the AEF had the highest sensitivity of85.2%, however, the 50th percentile of the AEF had the highest specificity of70.6%. For the mean value and corresponding percentiles of ΔAEF and rAEF, the 10th percentile of ΔAEF and rAEF had the highest AUC value of 0.788, with the cutoff value of − 0.034 and 0.952, respectively. The mean value, 75th percentile of ΔAEF, and the 75th percentile of rAEF had the highest sensitivity of 94.4%, and the 50th percentile of rAEF had the highest specificity of 82.4%. The 50th percentile of rAEF had the highest PPV value of 0.846, and the mean value of ΔAEF was the highest NPV value at 0.832. For the ∆HAP, the 10th percentile had the highest AUC value of 0.667, with a sensitivity and specificity of 64.8% and 67.6%, respectively. The 75th percentile of ∆HAP had the highest sensitivity of 88.9%, while the 25th and 50th percentile of ∆HAP had the highest specificity of 73.5%. The variance of PVP and the kurtosis of AEF also exhibited a statistical difference between IMCCs and HCCs, with AUCs of 0.665 and 0.636, respectively. The 25th percentile of HF_tumor_, and the 10th and 25th percentiles of ∆HF exhibited AUCs of 0.602, 0.638, and 0.606, respectively. The 25th percentile of HF had the highest sensitivity of 94.4%.

**Table 4:** ROC analysis of statistically significant parameters for the differentiation between HCCs and IMCCs.

	AUC	Sensitivity, %	Specificity, %	Cutoff value	PPV	NPV
AEF (mean)	0.715	0.796	0.647	0.536	0.782	0.666
AEF (0.1)	0.769	0.778	0.676	0.466	0.792	0.657
AEF (0.25)	0.766	0.852	0.676	0.492	0.807	0.742
AEF (0.5)	0.730	0.796	0.706	0.536	0.811	0.685
AEF (0.75)	0.678	0.704	0.676	0.573	0.775	0.590
AEF (0.9)	0.631	0.685	0.618	0.604	0.740	0.553
∆HAP (mean)	0.644	0.870	0.441	− 0.004	0.712	0.681
∆HAP (0.1)	0.666	0.648	0.676	0.001	0.761	0.547
∆HAP (0.25)	0.648	0.537	0.735	0.010	0.763	0.500
∆HAP (0.5)	0.639	0.537	0.735	0.013	0.763	0.500
∆HAP (0.75)	0.627	0.889	0.412	− 0.0008	0.706	0.701
∆AEF (mean)	0.726	0.944	0.441	0.00007	0.728	0.832
∆AEF (0.1)	0.788	0.870	0.618	− 0.034	0.783	0.750
∆AEF (0.25)	0.767	0.852	0.618	− 0.003	0.780	0.724
∆AEF (0.5)	0.745	0852	0.559	0.019	0.754	0.704
∆AEF (0.75)	0.705	0.944	0.412	0.023	0.718	0.822
∆AEF (0.9)	0.659	0.685	0.676	0.099	0.771	0.575
rAEF (mean)	0.726	0.593	0.794	1.140	0.821	0.551
rAEF (0.1)	0.788	0.833	0.647	0.952	0.789	0.709
rAEF (0.25)	0.766	0.852	0.618	0.994	0..780	0.724
rAEF (0.5)	0.746	0.611	0.824	1.131	0.846	0571
rAEF (0.75)	0.706	0.944	0.412	1.043	0.718	0.822
rAEF (0.9)	0.662	0.630	0.706	1.203	0.773	0.546
PVP (variance)	0.665	0.588	0.722	0.004	0.771	0.525
AEF (kurtosis)	0.636	0.882	0.352	3.293	0.684	0.653
HF (0.25)	0.602	0.944	0.265	0.108	0.671	0.749
∆HF (0.1)	0.638	0.722	0.529	− 0.178	0.709	0.545
∆HF (0.25)	0.606	0.778	0.412	− 0.165	0.678	0.539

*AUC* Area under the curve, *ROC* Receiver operating characteristic, *PPV* Positive predictive value, *NPV* Negative predictive value.

In conclusion, of all parameters, the 10th percentiles of ∆AEF and rAEF had the highest AUCs of 0.788; thus, indicating their abilities to provide differential diagnoses of all parameters. The mean value of ∆AEF, the 75th percentiles of ∆AEF and rAEF, and the 25th percentile of HF_tumor_ exhibited the highest sensitivity of 94.4%. The 50th percentile of rAEF exhibited the highest specificity of 82.4%. The 50th percentile of rAEF had the highest PPV value of 0.846, and the mean value of ∆AEF had the highest NPV value of 0.832.

## Discussion

Accurate differentiation between HCC and IMCC is challenging, but vital because their prognoses and treatments differ substantially. Tissue biopsy may not be routinely performed due to its invasiveness and concerns for procedure-related complications. Noninvasive modalities, such as contrast-enhanced CT and MRI are therefore the preferred methods for differentiating IMCC from HCC. In the present study, not only were the perfusion parameters derived from traditional triphasic CT scans, but the corresponding histogram analyses were also employed to further investigate the differentiation of the two tumors.

It is well-known that the main causes of liver cirrhosis were hepatitis viruses, mainly hepatitis B and C virus, and have been shown to be the principal risk factors for the occurrence of HCC^24^. Thus, a history of chronic hepatitis B or C infection promoted the development of HCC. However, hepatitis B and C virus infections were also risk factors for IMCC^25,26^, but our study showed no significant association between infection and IMCC. This result may be attributed to the small sample size. Our study findings also showed that patients with IMCC had lower a-fetoprotein (AFP) levels, and fewer liver cirrhosis cases compared to patients with HCC (*p* < 0.001). Such clinical features may provide a better differentiation of IMCCS from HCCs.

Perfusion computed tomography (PCT) is considered to be a prospective tool that could able to evaluate the hemodynamic changes in the liver and expand the role of CT from single morphological imaging to functional imaging. In Recent years, a simplified model of tumor blood perfusion derived from traditional triphasic CT scan has been developed and validated^21^. Lee et al.^27^ illuminated that perfusion parameters arise from traditional triphasic CT scans using the dual maximum slope model and there were no significant differences compared with routine PCT in liver and HCCs. As far as we know, this is the first study to use the perfusion parameters obtained from triphasic CT scans to differentiate the two tumors (IMCCs and HCCs). Our study demonstrated the values of AEF, ΔAEF, ΔHAP, rAEF for discriminating IMCCs from HCCs. All of the parameters mentioned above were significantly higher for HCCs than for IMCCs, which may indicate a relatively high hypervascularity in HCCs compared to IMCCs, and may also be indicative of the different pathological components of the tumor. We consider that the IMCC is peripherally rich in tumor cells with abundant fibrotic stroma and necrosis in the center, which may account for the enhancement patterns and relative hypo-vascularity^28^. Thus far, several studies^3,29–32^ have explored the vascularity and enhancement patterns of HCCs and IMCCs, and their findings were consistent with our study. The ΔAEF and rAEF both displayed a higher AUC (0.726) than other perfusion parameters, and were considered to be effective at distinguishing between IMCCs and HCCs. In addition, Hsu et al.^33^ previously revealed that perfusion parameters associated well with tumor survival and treatment responses in patients who received anti-angiogenic drugs. Therefore, we believe that this modality could also provide important information for the management of patients with IMCCs and HCCs. In the future, we will continue our investigations to improve its diagnostic abilities.

PCT is typically reported as a mean value. However, mean values do not illuminate the heterogeneity of tumors, especially the differences between IMCCs and HCCs, and thus, may not be optimal for tumor evaluations. The description of heterogeneity using histogram analyses has shown to be superior to mean values^34–37^. Zou et al.^36^ reported that analyses of the volumetric ADC histogram provided additional value to dynamic enhanced MRIs in differentiating IMCCs from HCCs. Asayama et al.^37^ also performed histogram analyses of ADC values to differentiate IMCCs from poorly differentiated HCCs. The result of our study showed that all the percentiles of AEF, ΔAEF, and ΔHAP were significantly higher in HCC cases than in IMCCs, and also identified significant differences in rAEF between HCCs and IMCCs. Our study results may indicate that there were significant differences in the heterogeneity between IMCCs and HCCs; a finding that was consistent with the previous study by Zou et al.^36^ that indicated that IMCC was more heterogeneous than HCC. Our results also suggested a relatively higher hypervascularity in HCCs than in IMCCs, which was also in agreement with a previous study by Zhao et al. and Choi et al.^38,39^. The 10th percentiles of the ΔAEF and rAEF showed the highest AUC of 0.788, which indicated that those factors had the best power to discriminate IMCCs from HCCs.

In the present study, significant differences were observed in the kurtosis and skewness between IMCCs and HCCs. However, kurtosis and skewness may be difficult to obtain and interpret^40^. Histogram kurtosis and skewness can be attributed to the asymmetric shape of the corresponding perfusion parameters distribution. This result may also be due to the heterogeneous differences between the two tumors. Regarding the sensitivity and specificity of the statistically significant variables in differentiating between HCCs and IMCCs, the mean AEF value, and the 75th percentiles of the ΔAEF and the rAEF exhibited the highest sensitivity of 94.4%. In addition, the 50th percentile of rAEF had the highest specificity of 82.4% for the differentiation of histological type of the two tumor types. Collectively, these results reflected that the discriminating ability of perfusion parameters and corresponding histogram parameters of AEF was superior to all other parameters.

Several limitations to our study need to be recognized. First, selection bias could not be completely avoided in this retrospective study. Second, the size of patient included was relatively small, especially the number of IMCC cases. Thus, further study with larger IMCCs population need to be conducted to enhance the statistical power. Third, most tumors in our study were of a relatively large size, while small HCCs or IMCCs more frequently appear as atypical lesions than larger ones, and therefore, there may have been a selection bias. Forth, the ROIs of lesion are only to be drawn in a few planes, not in all tumor volumes of interest for the analysis of parameters. Thus, it may affect the accuracy of the results. Fifth, to make the results more rigorous and convicing, there needs some subgroup analysis. For example, hepatitis virus-negative and cirrhosis-negative, hepatitis virus-negative and cirrhosis-positive, and so on. Lastly, we did not classify HCCs by histological grades which would affect the accuracy of the results, and we did not compare the performance between our study and other radiological and/or clinical diagnostic algorithms. We will further confirm its diagnostic efficiency compared with other methods in the future.

In conclusion, in this paper, we proposed liver perfusion parameters and corresponding histogram parameters for classifying two types of liver cancer, namely HCC and IMCC, from traditional triphasic CT scans. The 10th percentiles of the ΔAEF and rAEF exhibited the best differential power for preventing the misidentification of IMCC as HCC. The mean value of the AEF, and the 75th percentiles of ΔAEF and rAEF showed the highest sensitivity of 94.4%. However, the 50th percentile of rAEF had a highest specificity of 82.4%. Therefore, the results of this paper provided a quantitative, non-invasive method to facilitate the differentiation of IMCCs from HCCs.
